# Ethnicity-based classifications and medical genetics: One Health approaches from a Western Pacific perspective

**DOI:** 10.3389/fgene.2022.970549

**Published:** 2022-09-06

**Authors:** Hisham A. Edinur, Siti Nor A. Mat-Ghani, Geoffrey K. Chambers

**Affiliations:** ^1^ School of Health Sciences, Universiti Sains Malaysia, Kelantan, Malaysia; ^2^ School of Biological Sciences, Victoria University of Wellington, Wellington, New Zealand

**Keywords:** Austronesian, population genetics, admixture, ethnicity, One Health

## Abstract

A new era presently dawns for medical genetics featuring individualised whole genome sequencing and promising personalised medical genetics. Accordingly, we direct readers attention to the continuing value of allele frequency data from Genome-Wide Association Surveys (GWAS) and single gene surveys in well-defined ethnic populations as a guide for best practice in diagnosis, therapy, and prescription. Supporting evidence is drawn from our experiences working with Austronesian volunteer subjects across the Western Pacific. In general, these studies show that their gene pool has been shaped by natural selection and become highly diverged from those of Europeans and Asians. These uniquely evolved patterns of genetic variation underlie contrasting schedules of disease incidence and drug response. Thus, recognition of historical bonds of kinship among Austronesian population groups across the Asia Pacific has distinct public health advantages from a One Health perspective. Other than diseases that are common among them like gout and diabetes, Austronesian populations face a wide range of climate-dependent infectious diseases including vector-borne pathogens as they are now scattered across the Pacific and Indian Oceans. However, we caution that the value of genetic survey data in Austronesians (and other groups too) is critically dependent on the accuracy of attached descriptive information in associated metadata, including ethnicity and admixture.

## Introduction

Recent years have seen an explosion in the amount of human genetic data available to public health investigators. However, this information has been collected in many different places and for many different reasons. The true value of such abundance can only be captured by comparative analyses. To some extent, access to human genetic data are facilitated by the creation of open access databases such as the Allele Frequency Net Database [AFND; http://www.allelefrequencies.net/), or gnomAD, (https://gnomad.broadinstitute.org/)]. However, applications of these resources are strictly related to the completeness of associated metadata to individual records.

**FIGURE 1:**
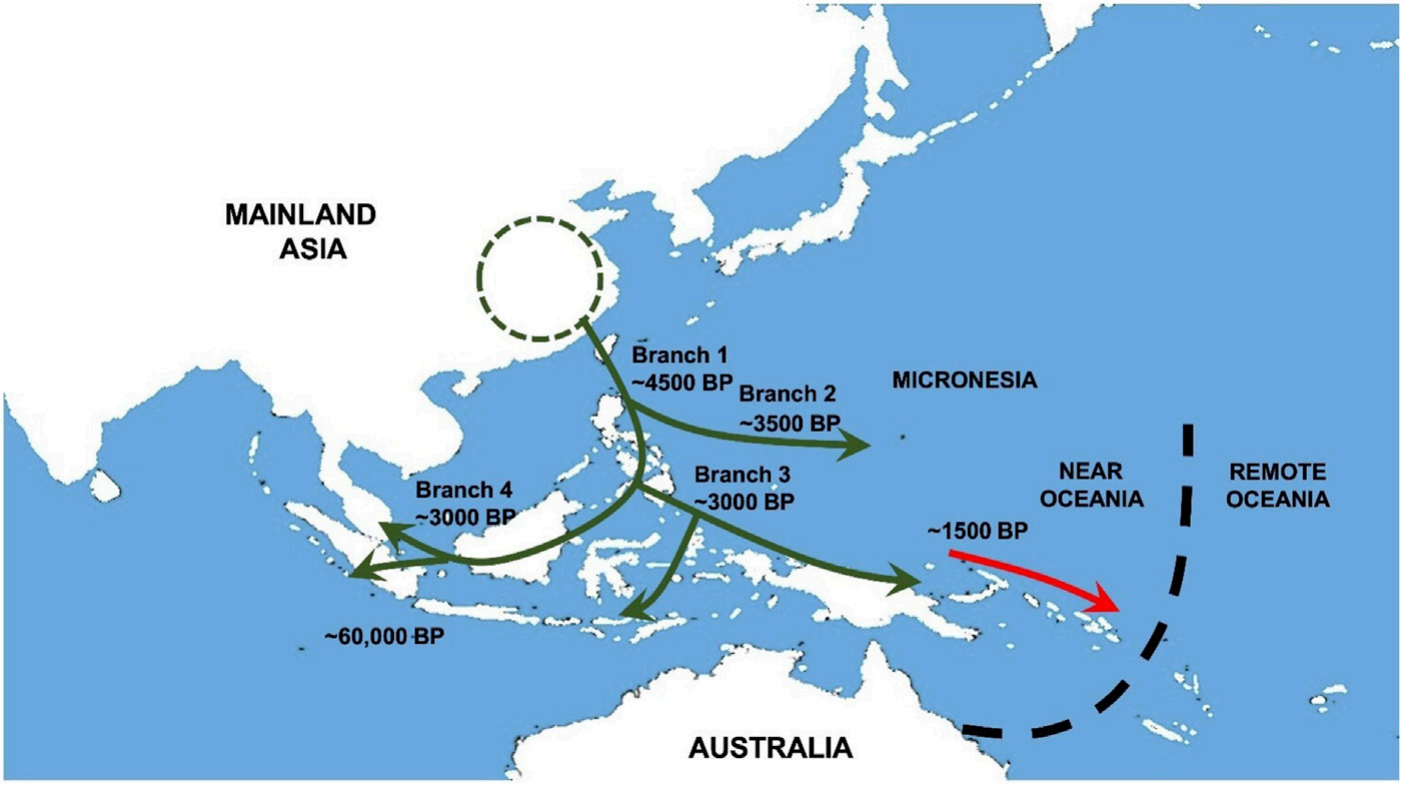
Austronesian population movements in Southeast Asia and Oceania. Early movements of Austronesians (green arrow) into the Southeast Asia region reach Philippines, Borneo, Indonesia and Near Oceania approximately 4500 to 1500 BP. Admixture between Austronesians and Papuans gave rise to Proto-Polynesians in northern coastal Papua New Guinea before their descendants (red arrow) migrated further out to Remote Oceania.

In this article, we describe and discuss what we have termed “The Austronesian Model” as it relates to medical genetics and public health in the peoples of the Western Pacific region. This model arises principally from our long-standing experience in immunogenetic surveys of Malay and Māori. Our genetic surveys and those of many others have led to a detailed picture of Austronesian population history and ancestral relationships (see Chambers and Edinur, 2021). This remarkable episode of human expansion from Southeast Asia to Melanesia and Polynesia (Figure 1) has left related groups of indigenous people spread halfway around the globe. Although they have a recent extensively shared heritage, they are by no means all identical. Hence, although there may well be a common interest in medical genetic information due to their shared ancestry, it requires careful interpretation due to recent sociocultural factors. Equally, individual patients should always be viewed as individuals and not simply taken to be representative of their ethnic group due to the possibility of recent admixture. Finally, ethnically coded databases must be annotated in much the same way, because it is never a given that they are homogeneous. Below, we examine all of these features in greater depth taking our examples from the indigenous aboriginal hill-tribes of Taiwan and their many descendants.

### A short history of the Austronesian peoples

Starting from around 5,000 years ago, groups of ancient farmers spread southwards from Taiwan (see Figure 1) and/or other locations in Southeast Asia to reach the Philippines and Borneo–Branch 1 (see Bellwood et al., 2011). Their lineage split with one group (Branch 4) going west to Malaysia, Indonesia and far out across the Indian Ocean to Madagascar. Another group (Branch 3) travelled across the north of the Papuan mainland to the Bismarck Archipelago, the probable birthplace of Lapita culture. This cultural complex was transported *via* Vanuatu to several nearby groups of Oceanic islands including Fiji, Tonga, and Samoa. The diaspora paused here, and the original pure Austronesian oceanic settlers were replaced by early Polynesian people from the wider Papuan region who were now Austronesians (70%) admixed with Australomelanesians (30%) and later still by Melanesians in Vanuatu, New Caledonia, and Fiji. Subsequent voyaging populated the Marquesas Island group in remote Oceania and diffused to fill up other sites in the so-called Polynesian Triangle. An extended account of these events can be found in Chambers and Edinur (2021), together with a complete list of references to the original work on which this concise historical reconstruction is based.

This expansion phenomenon is impressive by any standard and was undertaken by people with no written language or metal technology. Today their descendants number around 380 million people spread across at least 33 nation states. Their shared heritage leads to a commonality of public health interests, but the fundamental genetic differences between Austronesians and Polynesians must be considered.

### The advantages of shared history

Probably nobody would deny that the Austronesians represent an extensive and diverse cultural group. Recognition of their historical bonds of kinship has distinct public health advantages from a One Health perspective. This implies that medical research findings from one Austronesian population (i.e., One Health approach and the importance of genetic ethnicity) can probably be applied to others (with all prudent caveats as explained below). This is valuable because some populations are only small and thus poorly served in terms of research effort in this area. In contrast, others, e.g., in Indonesia, are extensive and they may host large scale modern genomic surveys of health significance (e.g., see Natri et al., 2020). So new findings can be translated from such populations to others. Further, the sheer weight of Austronesian numbers as a whole could be brought to bear as considerable leverage to intensify medical research for their benefit. This is a particularly important consideration if Austronesians as a group are genetically differentiated from other such groups (and we shall argue below that they are distinct and have their own well-known spectrum of medical conditions of particular concern).

Perhaps surprisingly, the genetic distinctiveness of ethnic groups is not a given among all academic disciplines. Many social scientists (e.g., Fullwiley, 2008; Hartigan, 2008) follow foundational views such as those of Boas (1912) or DuBois (1915) and hold that race and ethnicity are human intellectual constructs versus biological realities. In maintaining this position today, they draw support from Lewontin’s (1972) classical analysis of the distribution of genetic variation within and between human ethnic groups. This first showed that the majority of such variation lies between individuals rather than between groups, a finding endorsed by many others. Despite the universal veracity of this fact, as Edwards (2003) points out, it misses the point. Geographic populations are now known to harbour significantly characteristic genetic variation that distinguishes them from others. Consider, for example, the multiplicity of single gene mutations that have led to the inherited persistence of lactase expression in northern European farming communities. Liebert et al. (2017) who list at least five allelic up-regulation lactase variants, and Rotival et al. (2021) give numerous other examples in humans plants and animals. The standard social science view is made manifest in the policies of the American Anthropological Association (from 1998) and the American Sociological Association (from 2003) and see Byrd and Hughey (2015) on “the ideological double helix of racial inequality”. According to some, this view now looks to be in danger of being run over by a juggernaut of new genomic data, the views of its most loyal defenders notwithstanding (Duster, 2015). For a review of debate, see Chambers (2017).

For those that do hold the mindset view that all people are more or less genetically homogeneous, then for them there is no point in asking patients questions about their ancestry and no utility served by compiling medical databases stratified by ethnic identity. In contrast for Austronesian people, as a whole, we do endorse this approach with an important reservation regarding the Austronesian/Polynesian split. However, we argue that together these two groups are significantly different from others and that this fact is of fundamental medical significance. In particular, it is clear that, strictly in terms of their genetics, Austronesians are as different from Europeans as it is possible to be. Three general aspects of these differences can serve as pointers and some special cases will be examined in greater detail below. First, Austronesian peoples have a spectrum of disease incidence which is different from that of Europeans including an elevated incidence of gout, coronary heart disease and type II diabetes (Sakaue et al., 2003; Cheng et al., 2004), but only rarely display the autoimmune diseases like multiple sclerosis that are common in Europeans (see Edinur et al., 2012; Edinur et al., 2013). Second, in terms of transfusion and transplant markers it will always be easier for a Polynesian patient to find a matching donor from among their own ethnic group rather than from people of European ancestry (Edinur et al., 2015). Third, most “Big Pharma” researched medicines are sourced from Europe or the United States where they have undergone extensive clinical trials predominantly on people with European heritage (Glickman et al., 2009). They may also have been tested on relatively small numbers of volunteers recruited from ethnic minorities and sometimes may even carry disclaimers about their efficacy or safety for use in these groups (Hussain-Gambles, 2003).

### Limitations of the one health approach

The genetics of an organism are strictly related to ancestry and their expression is further shaped by their responses to environmental stress factors, weather, nutrition, medications, etc., (Natri et al., 2020). So, in the context of a One Health approach, the genetic identity of individuals becomes an essential factor to be considered alongside the others. In particular, we take the case of the Western Pacific population and discuss the pitfalls that a One Health study can have if genetic aspects of ethnicity are not considered. Providing acceptable names for ethnic groups is always difficult, e.g., see recent commentary by Popejoy (2021). In this regard, we have always tried to follow the field and use standard tags. These do not always age well, and we have sometimes found ourselves criticised by reviewers for using unfashionable names. Knowing exactly what these group names mean is important for comparisons between populations and for others to fairly assess the significance of research findings. Problems arise both at the group level *via* assumptions about uniformity and at the individual level *via* personal sense of identity and familial admixture history. There is a clear tension between social and biological definitions for ethnicity classification in research works. In our view “ethnicity” is a concept that sits at the exact interface between social customs and ancestry. Failure to properly recognise this fact lies at the heart of conflicts between social equity and patient welfare goals. Thus, we hold that workers in the field can gain only insights *via* a proper appreciation that “ethnicity” reflects both culture and ancestry. For instance, by recognizing that delivering social equity requires the use of social science-based definitions, such as the gold standard “self-declared ethnic affiliation”. Meaningful medical comparisons depend strictly on ancestry-based information, e.g., to assess the historical effect of natural selection on immune system genes (Nemat-Gorgani et al., 2014; Gonzalez-Galarza et al., 2020). Therefore, it is vital that those persons who wish to conduct meta-analyses of medical survey data should understand who their contributing data are sourced from. Archaic or broad scale descriptors such as “Asian” can lead to confusion in the same way that admixed groups, e.g., as in the US Census groups “Hispanic”, “Latino”, and “Oceanic” presently do. Such terms provide model examples of poorly constructed ethnic groups for medical research. Worse still, some investigators simply list populations by their geographic origin. This can lead to errors of interpretation. For instance, Friedlaender (2007) explains that across the New Britain and New Ireland archipelagos, northern coastal populations have more Austronesian genotypes and the interior and southern settlements more Australomelanesian genotypes.

The social histories of population groups in the Western Pacific are vastly different. For this reason alone they really should never all be combined, either as Asians, Oceanians, or Pacific Islanders etc., even for administrative convenience. These are seriously misleading classification schemes from the point of view of historical relationships. Arguably of even greater significance, is the fact that their various ages and genetic ancestries are also very different. The aboriginal people of Australia and Papuans are Australomelanesian and have been more or less distinct for > 40,000 years. In contrast, Polynesians (including Māori in New Zealand) only came into existence within the last 2 to 3,000 years and are of admixed Austronesian (∼70%) and Australomelanesian (∼30%) heritage—see Chambers and Edinur (2021) for more details. Human genetic diversity across the entire Western Pacific Region is further complicated by recent admixture between populations. For example, New Zealand (NZ) Māori contains many individuals with greater or lesser admixture with Europeans (and others). These groups (and the degree of admixture of individual patients) can only be assessed by careful interview technique (Edinur et al., 2012). Failure to properly examine for admixture can result in considerable biases in data and unreliable inferences. Worse, these may be perpetuated by their inclusion in public databases and by secondary references in the scientific literature. In short, one needs to be clear about just which population group a particular study represents so that others can fairly assess the significance of research findings.

Accommodating such considerations may not be entirely straightforward. For instance, Roberts (2021) protests that it is not reasonable for doctors to use just two adjustment factors for eGFR (effective glomerular filtration rates), African vs. non-African American, when the former self-identified group contains many admixed individuals. In this case it is both reasonable and justified to differentiate patients into two such loosely defined ethnic classes because this is done based on empirical data. In contrast this objection makes an important point about precision but is not justified because these factors already have an allowance for admixture built into them. This view is based on a self-identification process whereby admixed individuals are largely swept up into the African group. A more refined scale of correction factors could only be obtained *via* the administration of detailed volunteer ethnicity questionnaires and could only be applied to patients whose admixture fraction is known. It is doubtful if this would represent a practical proposition.

The goal of One Health is to achieve optimal healthy outcomes *via* a collaborative transdisciplinary approach by considering associations between living things including human and microbes and their surrounding environment (Centers for Disease Control and Prevention (CDC), 2021). In short, all people should expect to enjoy access to good quality, and affordable health care which is delivered in a culturally sensitive manner and is fully efficacious. This is to be achieved by increased medical research effort shared *via* open access publication. However, it is always to be remembered that people are not the same everywhere and that conditions vary across different regions of the world. In this context it is important to note that most Austronesian people currently live in low- and middle-income countries as either majority or minority groups. As described earlier, Austronesian people across the entire Asia-Pacific region have several health concerns in common, such as a high prevalence of gout and diabetes and are often underrepresented in medical research. However, they do face a wide range of diseases including vector-borne infections as they live in many totally different environments (e.g., temperate for Māori and Polynesians versus tropical or sub-tropical climates in Southeast Asia and across Oceania). Hence shared ancestry in Austronesians can be used as a preliminary guideline for disease prevention, diagnosis and medical treatment for conditions that are common among them. Other healthcare initiatives developed elsewhere such access to effective vaccines for endemic diseases should be made widely available to affected Austronesian population groups but applied with all due caution (Siramaneerat and Agushybana, 2021).

### Some illustrative examples

The fact that Austronesian people are relatively homogeneous, but ancestrally differentiated from both Europeans and Australomelanesians (except for Polynesians see below) has important consequences in terms of diagnosis and prescription. Their unique spectrum of disorders can guide physicians regarding which conditions and genetic markers they might look out for.

This concept is illustrated well by the variant rs2285666-A allele located in the *ACE2* gene. This has been shown to reduce risk and severity of SARS-CoV-2 infection (Devaux et al., 2020; Srivastava et al., 2020; Wang et al., 2020). The rs2285666-A allele frequency is around 0.143–0.225 in Europeans and 0.330–1.000 in Asian populations; refer Supplementary Table S1. As we write, Italy has a high rate of SARS-CoV-2 infection (29,333 cases per 100,000 people), and a mortality rate of 0.0028% (as of 5th June 2022, data from https://coronavirus.jhu.edu/map.html; John Hopkins University and Medicine, 2022). In contrast, the Asian country of Malaysia has a lower infection rate (just 13,939 cases per 100,000 people) with an associated mortality rate of 0.0011% (John Hopkins University and Medicine, 2022; Ministry of Health, 2022). Therefore, the differences in rs2285666-A allele frequency might contribute to the higher infection and mortality rates seen among Italians. Another well-known example is the CYP3A5*1 allele which is associated with greater production of CYP3A5 gene product. This protein contributes to differences in tacrolimus bioavailability between individuals. Tacrolimus is a chemical compound used to prevent post-transplant organ rejection. Carriers for CYP3A5*1 alleles have an increased tacrolimus clearance and this may cause a higher risk of rejection among renal transplant recipients (Chen and Prasad, 2018). Acute rejection in renal transplant is significantly higher among African American patients as compared with non-African American patients (Gralla et al., 2014; Taber et al., 2015). This is related to the higher frequency of CYP3A5*1 in Africans than other populations; refer Supplementary Table S2. Therefore, dosing for tacrolimus should be given based on patients CYP3A5 genotype.

As pointed out above this type of strategy is best guided by taking a detailed family history to account for ancestry. For instance, when we first obtained DNA samples (with all due Ethics permits) from NZ volunteer subjects for medical research purposes, we found major differences between two sub-sets of data (i.e., those from full ancestry vs. admixed heritage Māori). These two types in roughly equal proportions and allele frequencies recorded for the latter were approximately intermediate between those for the former and European reference data. This observation was taken to indicate that the admixture fraction in the contemporary population was around 0.50.

Hajar et al. (2020) have recently examined the interface between population genetics and human health as it relates to Austronesian populations. For example, they point to the significance of blood group loci as important markers of transfusion success including Kidd Jk (a-b+) and Duffy Fy (a+b+) which are relatively common in this ethnic group but less common in others. On other fronts Hawkins (2021) has recently examined the role of maladaptation to “European” nutrition as a potential root cause of metabolic syndrome in NZ Māori; Grinlinton et al. (2022) report elevated levels of Phyllodes Tumor in Māori and Pasifika and Bukhari et al. (2021) report reduced prevalence of neuromyelitis optica spectrum disorder and multiple sclerosis in both Māori plus Australian Aboriginal and Torres Strait Islander populations.

## Conclusion

We have discussed the importance that correct identification and use of population ancestry and structure can have on the reliability of One Health studies and approaches for improved understanding and treatment of medical conditions in Austronesian people. This unites a very large number of people spread across a vast geographic region and resident in many different political administrations in a search for solutions to shared problems. We note that future utility of this conceptual advance is conditioned to the extent that it can be informed by deeper understanding of population ancestry and structure. In particular, we point to the existence of clearly differentiated sub-groups such as Polynesians among the wider Austronesian ethnic group and the potential for recent admixture to add noise to otherwise promising genetic signals.

We recommend that practical application of these principles in relation to individual patients should be applied with caution so as to avoid discrimination. General properties which apply to populations do not necessarily apply to each individual that makes up such populations. For example, many Polynesian subjects are slow metabolisers of nicotine and may need large-sized or higher-dose patches to aid their transition to abstinence from tobacco consumption. These should not be given out automatically because not all Polynesians are slow metabolisers and slow metabolisers are also found among other ethnic groups (Lea et al., 2005). It is just more likely that such individuals may be encountered among Polynesian patients. Information of this type should properly be used only as a pointer to aid diagnosis or to suggest which medications to prescribe first for any given condition. This strategy will be further supported by detailed knowledge of their family history.

The medical world now stands at the dawn of the genomic era and eagerly awaits the advent of genuine personalised medicine. As an example of how this might work, we point to the study by Shankar et al. (2022). Future prospects for a genome-level One Health approach are well illustrated by their recent pharmacogenomic study involving Tiwi Islanders (from Northern Territory, Australia). This survey identified 22 significant genetic markers that can be screened in patients before prescribing pharmaceuticals used to alleviate common disorders such as chronic kidney disease. This type of information leads to 18 clinically actionable guidelines. Equally the results from other studies show how population history can set limits on the outreach of such benefits. Data from the 1000 Genomes Study show as many as 53% of rare variants detected by them were only found in individual populations, and 17% of low-frequency variants reside exclusively in single ancestry groups (see Larson et al., 2015 and McKenna et al., 2010 for other details). It is thus appropriate to recommend the use of genetic ancestry or other self-identification mechanisms that better reflect ancestry-based information in medical treatment and research. We argue that this will not obscure the value of a One Health approach for Austronesians but will likely complement it. Similar views have previously been expressed by Borrell et al. (2021) and we agree with their estimation that “… the epidemiologic importance of race/ethnicity will never disappear” even with the advent of widely available personalised medicine. We may now turn our attention considering how best to make use of medical genetic data collected across the Western Pacific Region.

## References

[B1] Adler G. Łoniewska B. Parczewski M. Kordek A. Ciechanowicz A. (2009). Frequency of common CYP3A5 gene variants in healthy Polish newborn infants. Pharmacol. Rep. 61, 947–951. 10.1016/s1734-1140(09)70154-9 PubMed Abstract | 10.1016/s1734-1140(09)70154-9 | Google Scholar 19904021

[B2] Arvanitidis K. Ragia G. Iordanidou M. Kyriaki S. Xanthi A. Tavridou A. (2007). Genetic polymorphisms of drug‐metabolizing enzymes CYP2D6, CYP2C9, CYP2C19 and CYP3A5 in the Greek population. Fundam. Clin. Pharmacol. 21, 419–426. 10.1111/j.1472-8206.2007.00510.x PubMed Abstract | 10.1111/j.1472-8206.2007.00510.x | Google Scholar 17635181

[B3] Asselta R. Paraboschi E. M. Mantovani A. Duga S. (2020). ACE2 and TMPRSS2 variants and expression as candidates to sex and country differences in COVID-19 severity in Italy. Aging (Albany NY) 12, 10087–10098. 10.18632/aging.103415 PubMed Abstract | 10.18632/aging.103415 | Google Scholar 32501810 PMC7346072

[B4] Azarpira N. Namazi S. Khalili A. Tabesh M. (2011). The investigation of allele and genotype frequencies of CYP3A5 (1*/3*) and P2Y12 (T744C) in Iran. Mol. Biol. Rep. 38, 4873–4877. 10.1007/s11033-010-0628-7 PubMed Abstract | 10.1007/s11033-010-0628-7 | Google Scholar 21153923

[B5] Badavi E. Safavi B. Jalali A. Shahriary G. M. Mohammadi-Asl J. Babaei J. (2015). Association of CYP3A4 and CYP3A5 polymorphisms with Iranian breast cancer patients. Egypt. J. Med. Hum. Genet. 16, 219–225. 10.1016/j.ejmhg.2015.03.004 10.1016/j.ejmhg.2015.03.004 | Google Scholar

[B6] Bains R. K. Kovacevic M. Plaster C. A. Tarekegn A. Bekele E. Bradman N. N. (2013). Molecular diversity and population structure at the Cytochrome P450 3A5 gene in Africa. BMC Genet. 14, 34. 10.1186/1471-2156-14-34 PubMed Abstract | 10.1186/1471-2156-14-34 | Google Scholar 23641907 PMC3655848

[B7] Balram C. Zhou Q. Cheung Y. B. Lee E. J. D. (2003). CYP3A5* 3 and* 6 single nucleotide polymorphisms in three distinct Asian populations. Eur. J. Clin. Pharmacol. 59, 123–126. 10.1007/s00228-003-0594-2 PubMed Abstract | 10.1007/s00228-003-0594-2 | Google Scholar 12756511

[B8] Bellwood P. Chambers G. K. Ross M. Hung H.-C. (2011). “Are ‘cultures’ inherited? Multidisciplinary perspectives on the origins of austronesian-speaking peoples prior to 1000 BC,” in Investigating archaeological cultures. Editors Roberts B. W. Linden M. V. (New York, NY: Springer), 321–354. 10.1007/978-1-4419-6970-5_16 | Google Scholar

[B9] Boas F. (1912). Changes in the bodily form of descendants of immigrants. Am. Anthropol. 14, 530–562. 10.1525/aa.1912.14.3.02a00080 10.1525/aa.1912.14.3.02a00080 | Google Scholar

[B10] Borrell L. N. Elhawary J. R. Fuentes-Afflick E. Witonsky J. Bhakta N. Wu A. H. B. (2021). Race and genetic ancestry in medicine - a time for reckoning with racism. N. Engl. J. Med. 384, 474–480. 10.1056/NEJMms2029562 PubMed Abstract | 10.1056/NEJMms2029562 | Google Scholar 33406325 PMC8979367

[B11] Bukhari W. Khalilidehkordi E. Mason D. F. Barnett M. H. Taylor B. V. Fabis-Pedrini M. (2021). NMOSD and MS prevalence in the Indigenous populations of Australia and New Zealand. J. Neurol. 269, 836–845. 10.1007/s00415-021-10665-9 PubMed Abstract | 10.1007/s00415-021-10665-9 | Google Scholar 34213614

[B12] Byrd W. C. Hughey M. W. (2015). Biological determinism and racial essentialism: The ideological double helix of racial inequality. Ann. Am. Acad. Pol. Soc. Sci. 661, 8–22. 10.1177/0002716215591476 10.1177/0002716215591476 | Google Scholar

[B13] Centers for Disease Control and Prevention (CDC) (2021). One health. Available at: https://www.cdc.gov/onehealth/index.html (Accessed June 5, 2022). Google Scholar

[B14] Chambers G. K. Edinur H. A. (2021). Reconstruction of the austronesian diaspora in the era of genomics. Hum. Biol. 92, 247–263. 10.13110/humanbiology.92.4.04 PubMed Abstract | 10.13110/humanbiology.92.4.04 | Google Scholar 34665569

[B15] Chambers G. K. (2017). Philosophy of race versus population genetics: Round 3. G. J. Anthropol. Res. 4, 26–36. 10.15379/2410-2806.2017.04.02.01 10.15379/2410-2806.2017.04.02.01 | Google Scholar

[B16] Chen L. Prasad G. V. R. (2018). CYP3A5 polymorphisms in renal transplant recipients: Influence on tacrolimus treatment. Pharmgenomics. Pers. Med. 11, 23–33. 10.2147/PGPM.S107710 PubMed Abstract | 10.2147/PGPM.S107710 | Google Scholar 29563827 PMC5846312

[B17] Cheng L. S.-C. Chiang S.-L. Tu H.-P. Chang S.-J. Wang T.-N. Ko A. M.-J. (2004). Genome-wide scan for gout in Taiwanese aborigines reveals linkage to chromosome 4q25. Am. J. Hum. Genet. 75, 498–503. 10.1086/423429 PubMed Abstract | 10.1086/423429 | Google Scholar 15252757 PMC1182028

[B18] Dally H. Bartsch H. Jäger B. Edler L. Schmezer P. Spiegelhalder B. (2004). Genotype relationships in the CYP3A locus in Caucasians. Cancer Lett. 207, 95–99. 10.1016/j.canlet.2003.12.011 PubMed Abstract | 10.1016/j.canlet.2003.12.011 | Google Scholar 15050738

[B19] Devaux C. A. Rolain J.-M. Raoult D. (2020). ACE2 receptor polymorphism: Susceptibility to SARS-CoV-2, hypertension, multi-organ failure, and COVID-19 disease outcome. J. Microbiol. Immunol. Infect. 53, 425–435. 10.1016/j.jmii.2020.04.015 PubMed Abstract | 10.1016/j.jmii.2020.04.015 | Google Scholar 32414646 PMC7201239

[B20] DuBois W. E. B. (1915). The negro. New York: Henry Holt. Google Scholar

[B21] Duster T. (2015). A post-genomic surprise: The molecular reinscription of race in science, law and medicine. Br. J. Sociol. 66, 1–27. 10.1111/1468-4446.12118 PubMed Abstract | 10.1111/1468-4446.12118 | Google Scholar 25789799

[B22] Edinur H. A. Chambers G. K. Dunn P. P. J. (2015). Recent developments in transplantation and transfusion medicine. Ann. Transpl. 20, 424–429. 10.12659/AOT.894003 PubMed Abstract | 10.12659/AOT.894003 | Google Scholar 26218888

[B23] Edinur H. A. Dunn P. P. J. Hammond L. Selwyn C. Brescia P. Askar M. (2013). HLA and MICA polymorphism in Polynesians and New Zealand maori: Implications for ancestry and health. Hum. Immunol. 74, 1119–1129. 10.1016/j.humimm.2013.06.011 PubMed Abstract | 10.1016/j.humimm.2013.06.011 | Google Scholar 23792058

[B24] Edinur H. A. Dunn P. P. J. Hammond L. Selwyn C. Velickovic Z. M. Lea R. A. (2012). Using HLA loci to inform ancestry and health in Polynesian and Maori populations. Tissue Antigens 80, 509–522. 10.1111/tan.12026 PubMed Abstract | 10.1111/tan.12026 | Google Scholar 23137322

[B25] Edwards A. W. F. (2003). Human genetic diversity: Lewontin’s fallacy. BioEssays 25, 798–801. 10.1002/bies.10315 PubMed Abstract | 10.1002/bies.10315 | Google Scholar 12879450

[B26] Floyd M. D. Gervasini G. Masica A. L. Mayo G. George A. L. Jr Bhat K. (2003). Genotype–phenotype associations for common CYP3A4 and CYP3A5 variants in the basal and induced metabolism of midazolam in European-and African-American men and women. Pharmacogenetics 13, 595–606. 10.1097/00008571-200310000-00003 PubMed Abstract | 10.1097/00008571-200310000-00003 | Google Scholar 14515058

[B27] Frere C. Cuisset T. Morange P.-E. Quilici J. Camoin-Jau L. Saut N. (2008). Effect of cytochrome p450 polymorphisms on platelet reactivity after treatment with clopidogrel in acute coronary syndrome. Am. J. Cardiol. 101, 1088–1093. 10.1016/j.amjcard.2007.11.065 PubMed Abstract | 10.1016/j.amjcard.2007.11.065 | Google Scholar 18394438

[B28] Friedlaender J. (2007). Genes, language, and culture history in the southwest pacific. New York: Oxford University Press. Google Scholar

[B29] Fukuen S. Fukuda T. Maune H. Ikenaga Y. Yamamoto I. Inaba T. (2002). Novel detection assay by PCR–RFLP and frequency of the CYP3A5 SNPs, CYP3A5*3 and *6, in a Japanese population. Pharmacogenetics 12, 331–334. 10.1097/00008571-200206000-00009 PubMed Abstract | 10.1097/00008571-200206000-00009 | Google Scholar 12042671

[B30] Fullwiley D. (2008). The biologistical construction of race: ‘admixture’ technology and the new genetic medicine. Soc. Stud. Sci. 38, 695–735. 10.1177/0306312708090796 PubMed Abstract | 10.1177/0306312708090796 | Google Scholar 19227818

[B31] Gervasini G. Vizcaino S. Gasiba C. Carrillo J. A. Benitez J. (2005). Differences in CYP3A5* 3 genotype distribution and combinations with other polymorphisms between Spaniards and other Caucasian populations. Ther. Drug Monit. 27, 819–821. 10.1097/01.ftd.0000186914.32038.a0 PubMed Abstract | 10.1097/01.ftd.0000186914.32038.a0 | Google Scholar 16306861

[B32] Glickman S. W. McHutchison J. G. Peterson E. D. Cairns C. B. Harrington R. A. Califf R. M. (2009). Ethical and scientific implications of the globalization of clinical research. N. Engl. J. Med. 360, 816–823. 10.1056/NEJMsb0803929 PubMed Abstract | 10.1056/NEJMsb0803929 | Google Scholar 19228627

[B33] Gonzalez-Galarza F. F. McCabe A. Santos E, J. M. D. Jones J. Takeshita L. Ortega-Rivera N. D. (2020). Allele frequency net database (AFND) 2020 update: Gold-standard data classification, open access genotype data and new query tools. Nucleic Acids Res. 48, D783–D788. 10.1093/nar/gkz1029 PubMed Abstract | 10.1093/nar/gkz1029 | Google Scholar 31722398 PMC7145554

[B34] Gralla J. Le C. N. Cooper J. E. Wiseman A. C. (2014). The risk of acute rejection and the influence of induction agents in lower‐risk African American kidney transplant recipients receiving modern immunosuppression. Clin. Transpl. 28, 292–298. 10.1111/ctr.12311 PubMed Abstract | 10.1111/ctr.12311 | Google Scholar 24476453

[B35] Grinlinton M. E. McGuiness M., J. Christie M. Oldfield R. Ramsaroop R. Moss D. (2022). Ethnic disparities in Phyllodes tumour in aotearoa New Zealand: A retrospective review. ANZ J. Surg. 92, 431–436. 10.1111/ans.17453 PubMed Abstract | 10.1111/ans.17453 | Google Scholar 35068031

[B36] Hajar C. G. N. Zefarina Z. Riffin N. Md.S. Tuan Mohammad T. H. Hassan M. N. Poonachi P. (2020). Extended blood group profiles for malays, Chinese and Indians in peninsular Malaysia. Egypt. J. Med. Hum. Genet. 21, 51. 10.1186/s43042-020-00096-y 10.1186/s43042-020-00096-y | Google Scholar

[B37] Hartigan J. Jr (2008). Is race still socially constructed? The recent controversy over race and medical genetics. Sci. Cult. (Lond). 17, 163–193. 10.1080/09505430802062943 10.1080/09505430802062943 | Google Scholar

[B38] Hawkins M. (2021). Were warriors once low carb? Commentary on New Zealand Māori nutrition and anthropometrics over the last 150 years. J. Prim. Health Care 13, 106–111. 10.1071/HC20129 PubMed Abstract | 10.1071/HC20129 | Google Scholar 34620290

[B39] He Y. Yang W. Liu S. Gan L. Zhang F. Mu C. (2017). Interactions between angiotensin-converting enzyme-2 polymorphisms and high salt intake increase the risk of hypertension in the Chinese Wa population. Int. J. Clin. Exp. Pathol. 10, 11159–11168. PubMed Abstract | Google Scholar 31966466 PMC6965882

[B40] Hilli J. Rane A. Lundgren S. Bertilsson L. Laine K. (2007). Genetic polymorphism of cytochrome P450s and P‐glycoprotein in the Finnish population. Fundam. Clin. Pharmacol. 21, 379–386. 10.1111/j.1472-8206.2007.00494.x PubMed Abstract | 10.1111/j.1472-8206.2007.00494.x | Google Scholar 17635176

[B41] Hu Y.-F. He J. Chen G.-L. Wang D. Liu Z.-Q. Zhang C. (2005). CYP3A5* 3 and CYP3A4* 18 single nucleotide polymorphisms in a Chinese population. Clin. Chim. Acta. 353, 187–192. 10.1016/j.cccn.2004.11.005 PubMed Abstract | 10.1016/j.cccn.2004.11.005 | Google Scholar 15698606

[B42] Hussain-Gambles M. (2003). Ethnic minority under-representation in clinical trials. J. Health Organ. Manag. 17, 138–143. 10.1108/14777260310476177 PubMed Abstract | 10.1108/14777260310476177 | Google Scholar 12916177

[B43] Itoyama S. Keicho N. Hijikata M. Quy T. Phi N. C. Long H. T. (2005). Identification of an alternative 5′‐untranslated exon and new polymorphisms of angiotensin‐converting enzyme 2 gene: Lack of association with SARS in the Vietnamese population. Am. J. Med. Genet. A 136, 52–57. 10.1002/ajmg.a.30779 PubMed Abstract | 10.1002/ajmg.a.30779 | Google Scholar 15937940 PMC7138097

[B44] Jakovski K. Kapedanovska-Nestorovska A. Labacevski N. Dimovski A. J. (2012). Frequency of the most common CYP3A5 polymorphisms in the healthy population of the Republic of Macedonia. Maced. Pharm. Bull. 58, 25–30. 10.33320/maced.pharm.bull.2012.58.003 10.33320/maced.pharm.bull.2012.58.003 | Google Scholar

[B45] Jin T. Zhang P. Shi X. Ouyang Y. He N. He X. (2016). Genetic analysis of drug‐metabolizing enzyme CYP3A5 polymorphisms in Tibetans in China. Int. J. Clin. Exp. Pathol. 9, 3717–3725. Google Scholar

[B46] John Hopkins University and Medicine (2022). COVID-19 dashboard. Available at: https://coronavirus.jhu.edu/map.html (Accessed June 5, 2022). Google Scholar

[B47] King B. P. Leathart J. B. S. Mutch E. Williams F. M. Daly A. K. (2003). CYP3A5 phenotype‐genotype correlations in a British population. Br. J. Clin. Pharmacol. 55, 625–629. 10.1046/j.1365-2125.2003.01798.x PubMed Abstract | 10.1046/j.1365-2125.2003.01798.x | Google Scholar 12814460 PMC1884247

[B48] Kudzi W. Dodoo A. N. Mills J. J. (2010). Genetic polymorphisms in MDR1, CYP3A4 and CYP3A5 genes in a Ghanaian population: A plausible explanation for altered metabolism of ivermectin in humans? BMC Med. Genet. 11, 111–118. 10.1186/1471-2350-11-111 PubMed Abstract | 10.1186/1471-2350-11-111 | Google Scholar 20630055 PMC3161347

[B49] Larson J. L. Silver A. J. Chan D. Borroto C. Spurrier B. Silver L. M. (2015). Validation of a high resolution NGS method for detecting spinal muscular atrophy carriers among phase 3 participants in the 1000 Genomes Project. BMC Med. Genet. 16, 100. 10.1186/s12881-015-0246-2 PubMed Abstract | 10.1186/s12881-015-0246-2 | Google Scholar 26510457 PMC4625734

[B50] Lea R. A. Benowitz N. Green M. Fowles J. Vishvanath A. Dickson S. (2005). Ethnic differences in nicotine metabolic rate among New Zealanders. N. Z. Med. J. 118, U1773. PubMed Abstract | Google Scholar 16372023

[B51] Lewontin R. C. (1972). “The apportionment of human diversity,” in Evolutionary biology. Editors Dobzhansky T. Hecht M. K. Steere W. C. (New York, NY: Springer), 381–398. 10.1007/978-1-4684-9063-3_14 | Google Scholar

[B52] Lieb W. Graf J. Götz A. König I. R. Mayer B. Fischer M. (2006). Association of angiotensin-converting enzyme 2 (ACE2) gene polymorphisms with parameters of left ventricular hypertrophy in men. Results of the MONICA Augsburg echocardiographic substudy. J. Mol. Med. 84, 88–96. 10.1007/s00109-005-0718-5 PubMed Abstract | 10.1007/s00109-005-0718-5 | Google Scholar 16283142

[B53] Liebert A. López S. Jones B. L. Montalva N. Gerbault P. Lau W. (2017). World-wide distributions of lactase persistence alleles and the complex effects of recombination and selection. Hum. Genet. 136, 1445–1453. 10.1007/s00439-017-1847-y PubMed Abstract | 10.1007/s00439-017-1847-y | Google Scholar 29063188 PMC5702378

[B54] Liu C. Li Y. Guan T. Lai Y. Shen Y. Zeyaweiding A. (2018). ACE2 polymorphisms associated with cardiovascular risk in Uygurs with type 2 diabetes mellitus. Cardiovasc. Diabetol. 17, 127. 10.1186/s12933-018-0771-3 PubMed Abstract | 10.1186/s12933-018-0771-3 | Google Scholar 30227878 PMC6142339

[B55] Lozano-Gonzalez K. Padilla-Rodríguez E. Texis T. Gutiérrez M. N. Rodríguez-Dorantes M. Cuevas-Córdoba B. (2020). Allele frequency of ACE2 intron variants and its association with blood pressure. DNA Cell Biol. 39, 2095–2101. 10.1089/dna.2020.5804 PubMed Abstract | 10.1089/dna.2020.5804 | Google Scholar 33016778

[B56] McKenna A. Hanna M. Banks E. Sivachenko A. Cibulskis K. Kernytsky A. (2010). The genome analysis toolkit: A MapReduce framework for analyzing next-generation DNA sequencing data. Genome Res. 20, 1297–1303. 10.1101/gr.107524.110 PubMed Abstract | 10.1101/gr.107524.110 | Google Scholar 20644199 PMC2928508

[B57] Ministry of Health (MOH) (2022). COVID-19 Malaysia latest updates. Available at: https://covid-19.moh.gov.my/terkini (Accessed June 5, 2022). Google Scholar

[B58] Mirghani R. A. Sayi J. Aklillu E. Allqvist A. Jande M. Wennerholm A. (2006). CYP3A5 genotype has significant effect on quinine 3-hydroxylation in Tanzanians, who have lower total CYP3A activity than a Swedish population. Pharmacogenet. Genomics 16, 637–645. 10.1097/01.fpc.0000230411.89973.1b PubMed Abstract | 10.1097/01.fpc.0000230411.89973.1b | Google Scholar 16906018

[B59] Natri H. M. Bobowik K. S. Kusuma P. Darusallam C. C. Jacobs G. S. Hudjashov G. (2020). Genome-wide DNA methylation and gene expression patterns reflect genetic ancestry and environmental differences across the Indonesian archipelago. PLoS Genet. 16, e1008749. 10.1371/journal.pgen.1008749 PubMed Abstract | 10.1371/journal.pgen.1008749 | Google Scholar 32453742 PMC7274483

[B60] Nemat-Gorgani N. Edinur H. A. Hollenbach J. A. Traherne J. A. Dunn P. P. J. Chambers G. K. (2014). KIR diversity in Māori and Polynesians: Populations in which HLA-B is not a significant KIR ligand. Immunogenetics 66, 597–611. 10.1007/s00251-014-0794-1 PubMed Abstract | 10.1007/s00251-014-0794-1 | Google Scholar 25139336 PMC4198482

[B61] Novelli A. Biancolella M. Borgiani P. Cocciadiferro D. Colona V. L. D’Apice M. R. (2020). Analysis of ACE2 genetic variants in 131 Italian SARS-CoV-2-positive patients. Hum. Genomics 14, 29. 10.1186/s40246-020-00279-z PubMed Abstract | 10.1186/s40246-020-00279-z | Google Scholar 32917283 PMC7483483

[B62] Ota T. Kamada Y. Hayashida M. Iwao-Koizumi K. Murata S. Kinoshita K. (2015). Combination analysis in genetic polymorphisms of drug-metabolizing enzymes CYP1A2, CYP2C9, CYP2C19, CYP2D6 and CYP3A5 in the Japanese population. Int. J. Med. Sci. 12, 78–82. 10.7150/ijms.10263 PubMed Abstract | 10.7150/ijms.10263 | Google Scholar 25552922 PMC4278879

[B63] Park S. Y. Kang Y. S. Jeong M. S. Yoon H. K. Han K. O. (2008). Frequencies of CYP3A5 genotypes and haplotypes in a Korean population. J. Clin. Pharm. Ther. 33, 61–65. 10.1111/j.1365-2710.2008.00879.x PubMed Abstract | 10.1111/j.1365-2710.2008.00879.x | Google Scholar 18211618

[B64] Popejoy A. B. (2021). Too many scientists still say Caucasian. Nature 596, 463. 10.1038/d41586-021-02288-x 10.1038/d41586-021-02288-x | Google Scholar

[B65] Roberts D. E. (2021). Abolish race correction. Lancet 397, 17–18. 10.1016/S0140-6736(20)32716-1 PubMed Abstract | 10.1016/S0140-6736(20)32716-1 | Google Scholar 33388099

[B66] Rotival M. Cossart P. Quintana-Murci L. (2021). Reconstructing 50, 000 years of human history from our DNA: Lessons from modern genomics. C. R. Biol. 344, 177–187. 10.5802/crbiol.55 PubMed Abstract | 10.5802/crbiol.55 | Google Scholar 34213855

[B67] Sajib A. A. Aziz R. Shariar S. Khan A. Akhteruzzaman S. (2019). Multiplex allele-specific PCR to determine genotypes at statin metabolizing SNP loci-rs1135840 and rs776746. J. Appl. Biol. Sci. 13, 96–102. Google Scholar

[B68] Sakaue M. Fuke Y. Katsuyama T. Kawabata M. Taniguchi H. (2003). Austronesian-speaking people in Papua New Guinea have susceptibility to obesity and type 2 diabetes. Diabetes Care 26, 955–956. 10.2337/diacare.26.3.955-a PubMed Abstract | 10.2337/diacare.26.3.955-a | Google Scholar 12610072

[B69] Semiz S. Dujic T. Ostanek B. Prnjavorac B. Bego T. Malenica M. (2011). Analysis of CYP3A4* 1B and CYP3A5* 3 polymorphisms in population of Bosnia and Herzegovina. Med. Glas. 8, 84–89. PubMed Abstract | Google Scholar 21263403

[B70] Shankar A. J. Jadhao S. Hoy W. Foote S. J. Patel H. R. Scaria V. (2022). Pharmacogenomic analysis of a genetically distinct Indigenous population. Pharmacogenomics J. 22, 100–108. 10.1038/s41397-021-00262-4 PubMed Abstract | 10.1038/s41397-021-00262-4 | Google Scholar 34824386

[B71] Sinues B. Vicente J. Fanlo A. Vasquez P. Medina J. C. Mayayo E. (2007). CYP3A5* 3 and CYP3A4* 1B allele distribution and genotype combinations: Differences between Spaniards and central Americans. Ther. Drug Monit. 29, 412–416. 10.1097/FTD.0b013e31811f390a PubMed Abstract | 10.1097/FTD.0b013e31811f390a | Google Scholar 17667794

[B72] Siramaneerat I. Agushybana F. (2021). Inequalities in immunization coverage in Indonesia: A multilevel analysis. Rural. Remote Health 21, 6348. 10.22605/rrh6348 PubMed Abstract | 10.22605/rrh6348 | Google Scholar 34432982

[B73] Srivastava A. Bandopadhyay A. Das D. Pandey R. K. Singh V. Khanam N. (2020). Genetic association of ACE2 rs2285666 polymorphism with COVID-19 spatial distribution in India. Front. Genet. 11, 564741. 10.3389/fgene.2020.564741 PubMed Abstract | 10.3389/fgene.2020.564741 | Google Scholar 33101387 PMC7545580

[B74] Strafella C. Caputo V. Termine A. Barati S. Gambardella S. Borgiani P. (2020). Analysis of ACE2 genetic variability among populations highlights a possible link with COVID-19-related neurological complications. Genes 11, 741. 10.3390/genes11070741 PubMed Abstract | 10.3390/genes11070741 | Google Scholar PMC7397291 32635188

[B75] Suarez-Kurtz G. Vargens D. D. Santoro A. B. Hutz M. H. de Moraes M. E. Pena S. D. J. (2014). Global pharmacogenomics: Distribution of CYP3A5 polymorphisms and phenotypes in the Brazilian population. PLoS One 9, e83472. 10.1371/journal.pone.0083472 PubMed Abstract | 10.1371/journal.pone.0083472 | Google Scholar 24427273 PMC3888384

[B76] Swart M. Skelton M. Wonkam A. Kannemeyer L. Chin’ombe N. Dandara C. (2012). CYP1A2, CYP2A6, CYP2B6, CYP3A4 and CYP3A5 polymorphisms in two Bantu-speaking populations from Cameroon and south Africa: Implications for global pharmacogenetics. Curr. Pharmacogenomics Person. Med. 10, 43–53. 10.2174/1875692111201010043 10.2174/1875692111201010043 | Google Scholar

[B77] Taber D. J. Gebregziabher M. G. Srinivas T. R. Chavin K. D. Baliga P. K. Egede L. E. (2015). African‐American race modifies the influence of tacrolimus concentrations on acute rejection and toxicity in kidney transplant recipients. Pharmacotherapy 35, 569–577. 10.1002/phar.1591 PubMed Abstract | 10.1002/phar.1591 | Google Scholar 26011276 PMC4534305

[B78] Vaarala M. H. Mattila H. Ohtonen P. Tammela T. L. J. Paavonen T. K. Schleutker J. (2008). The interaction of CYP3A5 polymorphisms along the androgen metabolism pathway in prostate cancer. Int. J. Cancer 122, 2511–2516. 10.1002/ijc.23425 PubMed Abstract | 10.1002/ijc.23425 | Google Scholar 18306354

[B79] Wang J. Xu X. Zhou X. Chen P. Liang H. Li X. (2020). Molecular simulation of SARS-CoV-2 spike protein binding to pangolin ACE2 or human ACE2 natural variants reveals altered susceptibility to infection. J. Gen. Virol. 101, 921–924. 10.1099/jgv.0.001452 PubMed Abstract | 10.1099/jgv.0.001452 | Google Scholar 32538738 PMC7654750

[B80] Wang S.-X. Tao T. Fu Z.-Q. Xie X.-Z. Hao W. Wang Y.-T. (2013). Polymorphisms of angiotensin-converting enzyme 2 gene confer a risk to lone atrial fibrillation in Chinese male patients. Chin. Med. J. 126, 4608–4611. PubMed Abstract | Google Scholar 24342297

[B81] Xu Y. Bao Q. He B. Pan Y. Zhang R. Mao X. (2012). Association of angiotensin I converting enzyme, angiotensin II type 1 receptor and angiotensin I converting enzyme 2 gene polymorphisms with the dyslipidemia in type 2 diabetic patients of Chinese Han origin. J. Endocrinol. Invest. 35, 378–383. 10.3275/7797 PubMed Abstract | 10.3275/7797 | Google Scholar 21670585

[B82] Yousef A.-M. Bulatova N. R. Newman W. Hakooz N. Ismail S. Qusa H. (2012). Allele and genotype frequencies of the polymorphic cytochrome P450 genes (CYP1A1, CYP3A4, CYP3A5, CYP2C9 and CYP2C19) in the Jordanian population. Mol. Biol. Rep. 39, 9423–9433. 10.1007/s11033-012-1807-5 PubMed Abstract | 10.1007/s11033-012-1807-5 | Google Scholar 22722998

