# Supplementary material for: Ethnicity-based classifications and medical genetics: One Health approaches from a Western Pacific perspective
Source: Front Genet. 2022 Sep 6;13:970549. doi: 10.3389/fgene.2022.970549 (PMC9485872; doi:10.3389/fgene.2022.970549)
Supplement: Supplementary file 1 [file DataSheet1.docx]

# Supplementary Tables

**Supplementary Table S1.** Allele frequencies of rs2285666 variants in the *ACE2* gene for selected population groups.

| Region | Population | Allelic frequency | | Reference |
| --- | --- | --- | --- | --- |
|  |  | **A** | **G** |  |
| American | Mexican Mestizos | 0.394 | 0.606 | (Lozano-Gonzalez et al., 2020) |
| Asian | Bangladesh Khumi | 0.500 | 0.500 | (Srivastava et al., 2020) |
|  | Bangladesh Marma | 0.750 | 0.250 | (Srivastava et al., 2020) |
|  | Bangladesh Tanchagya | 0.500 | 0.500 | (Srivastava et al., 2020) |
|  | Bangladeshi | 0.344 | 0.656 | (Srivastava et al., 2020) |
|  | Chinese | 0.765 | 0.236 | (Xu et al., 2012) |
|  | Chinese Han | 0.506 | 0.494 | (Wang et al., 2013) |
|  | Chinese Uygurs | 0.430 | 0.570 | (Liu et al., 2018) |
|  | Chinese Wa | 0.504 | 0.496 | (He et al., 2017) |
|  | Indian Assam_Mix | 0.510 | 0.490 | (Srivastava et al., 2020) |
|  | Indian Bengali | 0.554 | 0.446 | (Srivastava et al., 2020) |
|  | Indian Bihar_Mix | 0.575 | 0.425 | (Srivastava et al., 2020) |
|  | Indian Brahmins | 0.443 | 0.557 | (Srivastava et al., 2020) |
|  | Indian Brahmins and Kshatria | 0.460 | 0.540 | (Srivastava et al., 2020) |
|  | Indian Cochin_Jews and Mix Malyal | 0.875 | 0.125 | (Srivastava et al., 2020) |
|  | Indian Garo and Khasi | 0.756 | 0.244 | (Srivastava et al., 2020) |
|  | Indian Gujarati | 0.400 | 0.600 | (Srivastava et al., 2020) |
|  | Indian Gujjar | 0.586 | 0.414 | (Srivastava et al., 2020) |
|  | Indian Marathi_Parsi_Jews | 0.330 | 0.670 | (Srivastava et al., 2020) |
|  | Indian Nyshi_Bom_Mro_Oraon | 1.000 | 0.000 | (Srivastava et al., 2020) |
|  | Indian Ror | 0.550 | 0.450 | (Srivastava et al., 2020) |
|  | Indian Santhal_Munda_Oraon | 0.700 | 0.300 | (Srivastava et al., 2020) |
|  | Indian Tamil and Sri Lankan Tamil | 0.490 | 0.510 | (Srivastava et al., 2020) |
|  | Indian Telugu | 0.546 | 0.454 | (Srivastava et al., 2020) |
|  | Indian Tripuri | 1.000 | 0.000 | (Srivastava et al., 2020) |
|  | Vietnamese | 0.450 | 0.550 | (Itoyama et al., 2005) |
| European | German | 0.209 | 0.792 | (Lieb et al., 2006) |
|  | Italian Population 1 | 0.143 | 0.857 | (Strafella et al., 2020) |
|  | Italian Population 2 | 0.225 | 0.775 | (Novelli et al., 2020) |
|  | Italian Population 3 | 0.206 | 0.794 | (Asselta et al., 2020) |

**Supplementary Table S2.** Allele frequencies of *CYP3A5* variants in selected populations.

| Region | Population | Allelic frequency | | | | Reference |
| --- | --- | --- | --- | --- | --- | --- |
|  |  | *CYP3A5*1* | *CYP3A5*3* | *CYP3A5*6* | *CYP3A5*7* |  |
| American | European American | 0.130 | 0.870 | - | - | (Floyd et al., 2003) |
|  | African American | 0.721 | 0.279 | - | - | (Floyd et al., 2003) |
|  | Brazillian White | 0.150 | 0.820 | 0.020 | 0.010 | (Suarez-Kurtz et al., 2014) |
|  | Brazillian Brown | 0.260 | 0.680 | 0.050 | 0.040 | (Suarez-Kurtz et al., 2014) |
|  | Brazillian Black | 0.320 | 0.520 | 0.110 | 0.080 | (Suarez-Kurtz et al., 2014) |
|  | Central American | 0.237 | 0.763 | - | - | (Sinues et al., 2007) |
| European | British | 0.060 | 0.940 | - | - | (King et al., 2003) |
|  | Bosnian and Herzegovian | 0.068 | 0.932 | - | - | (Semiz et al., 2011) |
|  | Finnish Population 1 | 0.070 | 0.930 | - | - | (Vaarala et al., 2008) |
|  | Finnish Population 2 | 0.079 | 0.921 | - | - | (Hilli et al., 2007) |
|  | French | 0.107 | 0.893 | - | - | (Frere et al., 2008) |
|  | German | 0.062 | 0.938 | - | - | (Dally et al., 2004) |
|  | Northern Spanish | 0.086 | 0.914 | - | - | Sinues et al., 2007 |
|  | Greek | 0.057 | 0.943 | - | - | (Arvanitidis et al., 2007) |
|  | Macedonian | 0.078 | 0.922 | - | - | (Jakovski et al., 2012) |
|  | Polish | 0.060 | 0.940 | - | - | (Adler et al., 2009) |
|  | Spanish | 0.090 | 0.910 | - | - | (Gervasini et al., 2005) |
|  | Swedish | 0.070 | 0.930 | - | - | (Mirghani et al., 2006) |
| Asian | Bangladeshi | 0.360 | 0.640 | - | - | (Sajib et al., 2019) |
|  | Chinese | 0.222 | 0.778 | - | - | (Hu et al., 2005) |
|  | Chinese Tibetans | 0.516 | 0.484 | 0.000 | - | (Jin et al., 2016) |
|  | Iranian Population 1 | 0.174 | 0.826 | - | - | (Azarpira et al., 2011) |
|  | Iranian population 2 | 0.010 | 0.990 | - | - | (Badavi et al., 2015) |
|  | Japanese Population 1 | 0.238 | 0.762 | - | - | (Ota et al., 2015) |
|  | Japanese Population 2 | 0.233 | 0.768 | 0.000 | - | (Fukuen et al., 2002) |
|  | Jordanian | 0.072 | 0.926 | 0.002 | - | (Yousef et al., 2012) |
|  | Korean | 0.235 | 0.765 | 0.000 | 0.000 | (Park et al., 2008) |
|  | Singaporean Chinese | 0.250 | 0.760 | 0.000 | - | (Balram et al., 2003) |
|  | Singaporean Indians | 0.410 | 0.590 | 0.000 | - | (Balram et al., 2003) |
|  | Singaporean Malays | 0.390 | 0.610 | 0.000 | - | (Balram et al., 2003) |
| African | South African | 0.770 | 0.230 | - | - | (Swart et al., 2012) |
|  | Cameroonian Population 1 | 0.830 | 0.170 | - | - | (Swart et al., 2012) |
|  | Cameroonian Population 2 | 0.730 | 0.270 | - | - | (Bains et al., 2013) |
|  | Cameroonian Population 3 | 0.600 | 0.400 | - | - | (Bains et al., 2013) |
|  | Cameroonian Population 4 | 0.770 | 0.230 | - | - | (Bains et al., 2013) |
|  | Cameroonian Population 5 | 0.730 | 0.270 | - | - | (Bains et al., 2013) |
|  | Congolese | 0.800 | 0.200 | - | - | (Bains et al., 2013) |
|  | Ethiopian Population 1 | 0.350 | 0.650 | - | - | (Bains et al., 2013) |
|  | Ethiopian Population 2 | 0.330 | 0.670 | - | - | (Bains et al., 2013) |
|  | Ethiopian Population 3 | 0.710 | 0.290 | - | - | (Bains et al., 2013) |
|  | Ethiopian Population 4 | 0.510 | 0.490 | - | - | (Bains et al., 2013) |
|  | Ethiopian Population 5 | 0.350 | 0.650 | - | - | (Bains et al., 2013) |
|  | Ghanaian Population 1 | 0.890 | 0.110 | - | - | (Bains et al., 2013) |
|  | Ghanaian Population 2 | 0.810 | 0.190 | - | - | (Bains et al., 2013) |
|  | Ghanaian Population 3 | 0.780 | 0.220 | - | - | (Bains et al., 2013) |
|  | Ghanaian Population 4 | 0.850 | 0.150 | - | - | (Kudzi et al., 2010) |
|  | Malawi Population 1 | 0.850 | 0.150 | - | - | (Bains et al., 2013) |
|  | Malawi Population 2 | 0.830 | 0.170 | - | - | (Bains et al., 2013) |
|  | Malawi Population 3 | 0.890 | 0.110 | - | - | (Bains et al., 2013) |
|  | Malawi Population 4 | 0.850 | 0.150 | - | - | (Bains et al., 2013) |
|  | Malawi Population 5 | 0.820 | 0.180 | - | - | (Bains et al., 2013) |
|  | Moroccan | 0.200 | 0.800 | - | - | (Bains et al., 2013) |
|  | Mozambican | 0.840 | 0.160 | - | - | (Bains et al., 2013) |
|  | Nigerian | 0.870 | 0.130 | - | - | (Bains et al., 2013) |
|  | Senegalese Population 1 | 0.790 | 0.210 | - | - | (Bains et al., 2013) |
|  | Senegalese Population 2 | 0.750 | 0.250 | - | - | (Bains et al., 2013) |
|  | Southern African | 0.740 | 0.260 | - | - | (Bains et al., 2013) |
|  | Sudanese Population 1 | 0.400 | 0.600 | - | - | (Bains et al., 2013) |
|  | Sudanese Population 2 | 0.550 | 0.450 | - | - | (Bains et al., 2013) |
|  | Tanzanian Population 1 | 0.740 | 0.260 | - | - | (Bains et al., 2013) |
|  | Tanzanian Population 2 | 0.510 | 0.190 | 0.180 | 0.120 | (Mirghani et al., 2006) |
|  | Ugandan | 0.960 | 0.040 | - | - | (Bains et al., 2013) |
|  | Zimbabwean Population 1 | 0.870 | 0.130 | - | - | (Bains et al., 2013) |
|  | Zimbabwean Population 2 | 0.840 | 0.160 | - | - | (Bains et al., 2013) |
